# Population Estimation Methods for Free-Ranging Dogs: A Systematic Review

**DOI:** 10.1371/journal.pone.0144830

**Published:** 2015-12-16

**Authors:** Vinícius Silva Belo, Guilherme Loureiro Werneck, Eduardo Sérgio da Silva, David Soeiro Barbosa, Claudio José Struchiner

**Affiliations:** 1 Departamento de Endemias Samuel Pessoa, Fundação Oswaldo Cruz, Rio de Janeiro, RJ, Brasil; 2 Departamento de Epidemiologia - Instituto de Medicina Social, Universidade do Estado do Rio de Janeiro, Rio de Janeiro, Brasil; 3 Campus Centro-Oeste Dona Lindu, Universidade Federal de São João del Rei, Divinópolis, Minas Gerais, Brasil; University of Portsmouth, UNITED KINGDOM

## Abstract

The understanding of the structure of free-roaming dog populations is of extreme importance for the planning and monitoring of populational control strategies and animal welfare. The methods used to estimate the abundance of this group of dogs are more complex than the ones used with domiciled owned dogs. In this systematic review, we analyze the techniques and the results obtained in studies that seek to estimate the size of free-ranging dog populations. Twenty-six studies were reviewed regarding the quality of execution and their capacity to generate valid estimates. Seven of the eight publications that take a simple count of the animal population did not consider the different probabilities of animal detection; only one study used methods based on distances; twelve relied on capture-recapture models for closed populations without considering heterogeneities in capture probabilities; six studies applied their own methods with different potential and limitations. Potential sources of bias in the studies were related to the inadequate description or implementation of animal capturing or viewing procedures and to inadequacies in the identification and registration of dogs. Thus, there was a predominance of estimates with low validity. Abundance and density estimates carried high variability, and all studies identified a greater number of male dogs. We point to enhancements necessary for the implementation of future studies and to potential updates and revisions to the recommendations of the World Health Organization with respect to the estimation of free-ranging dog populations.

## Introduction

In Ecology, the term “population” defines a group of organisms of one species that interbreed and live in the same place at the same time [1]. Plenty of estimates of abundance have been obtained, especially for populations of wild animals, for example, elephants [2], frogs [3], birds [4], wales [5], insects [6] and foxes [7]. In relation to dogs, although a great percentage of them are domiciliary and restricted animals, there is still a great percentage of animals continuously circulating on the street [8] forming specific population groups which are named free-roaming, stray, wandering, not domiciled or unrestricted. Even though publications with free roaming dogs were less frequent than with domiciliary dogs [9], non-restricted dogs also had their population size estimated in different studies [10].

Stray dogs can be considered the primary victims of irresponsible owners who reject their pets into the streets [10, 11]. The abandonment of dogs might be related to irresponsible breeding and religious, cultural and socioeconomic factors. The existence of free-roaming dogs is considered an important problem, not only for the animal welfare but also for Public Health [12, 13].

Issues related to non-domiciliary dogs refer to incidents such as bites, transmission of diseases to humans, damage to wildlife populations, accidents and pollution [14–17].

Adequate estimates of the size and of the characteristics of the population of free-roaming dogs are essential in planning and monitoring the effectiveness of strategies adopted for the control and for the welfare of the population [18, 19], for the management of risks associated with their presence [20] and to quantify the prevalence of zoonosis and other diseases present in these animals.

The methods used to estimate the abundance of domiciliary dogs, such as the assessment of veterinary records or sampling based on questionnaires applied to owners, are not useful to estimate the abundance of free-ranging dogs [21]. On the contrary, it is more appropriate to rely on the same methods developed for the estimation of abundance of the population of wild animals [9]. Nowadays, a wide variety of techniques can be applied, including indirect methods, sample surveys based on counts, lines, transects or points; radio telemetry and plenty of other different forms of capture and recapture [22, 23]. The development of such techniques continues to grow [24],but despite all the recent methodological advances, the WHO [25] and the World Animal Protection [26] recommend only four techniques to be used in the estimation of the abundance of unrestricted dogs: total or indirect counts, regression method, estimates of recaptures and the Beck method. Even though such methods have the advantage of being easier to understand and apply [19], in many cases, their premises [27] cannot be met in the studies of the dynamics of the population of free-roaming dogs, when the application of more appropriate techniques is required. Besides, it is necessary that the methods used be up to date with the advances made in this field of study [23, 28, 29].

A recent systematic review of methods used to estimate the size of restricted domiciliary dogs showed that these were, in general, considered questionable [30]. This limitation was due to a large number of biases and constraints found in most publications and highlighted the importance of a critical evaluation of the knowledge produced and used in the field of veterinary medicine [30]. Given the additional complexities involved in the estimation of the abundance of free-roaming dogs, and the need to choose the appropriate procedures, with known statistical properties and assumptions [22], we systematically review the literature on the estimation of the size of this population. In particular, we assess the methodological framework under which these estimates were carried out as well as the patterns obtained in the estimates in terms of gender, age, breed and socioeconomic factors.

## Materials and Methods

### Eligibility requirements

To be included in the systematic review, each of the studies must report at least one original assessment of the abundance, or of the density or of the ratio of humans by dogs of a free-ranging dog population. The definition of free-roaming dogs used was based on the criteria of the World Health Organization [25] as follows: i. family dogs (completely dependent, but semi-restricted); ii. neighborhood or community dogs (semi-dependent and unrestricted or semi-restricted); and iii. feral dogs (independent and unrestricted). There were no restrictions about the language of the publication.

We excluded studies published before 1980, modeling studies that did not report abundance estimates, reviews and studies in which the method used was not described in enough details to make it possible to understand the estimation process.

### Searches, selection and extraction of information from the publications

Searching strategies of publications were implemented during the month of November of 2013. Four different databases (Web of Science, Scopus, Ovid Cab Abstracts and ProQuest) and Google Scholar tool were used with the following search terms or their derivatives, depending on the "subject headings" of each base: (Dog*) OR (kennels) OR (canine) OR (canidae) AND ("Estimates") OR ("size") OR ("population*") OR ("dynamics") OR ("abundance") OR ("stray") OR ("demography"). The search was performed considering the occurrence of the terms only on the title of the publications. In addition, we asked for the contribution of experts on the topic.

Based on the titles and abstracts of the identified studies, we excluded those considered irrelevant given the inclusion criteria. After remission of duplicates, we analyzed the full texts of potentially relevant publications. In this phase, we selected the studies included in the present review.

The texts of publications with restricted access were obtained through the Oswaldo Cruz Foundation—Capes Portal. The articles not available via the portal were accessed through the Harvard Library Resource Sharing-Countway Library. Finally, in case of failure of the two previous strategies, we directly contacted the authors or the journal in which the study was published.

The extraction of information from complete texts was done by one of the review authors (VSB) and verified by the others. For each study, when available, the following information was recorded: year; place of execution; definition of free-roaming dog; method used for marking and identification of the dog; method used for observation and/or dog capture procedures; analytical procedure used to obtain the population size; calculated population size; density; male-female ratio; number of dogs by inhabitants of the area; age and breed.

### Organization and analysis of the quality of the collected information

We describe the number of publications analyzed by continents and countries. The studies were then divided into four groups considering the type of method used to obtain the estimate: i. Censuses or counts; ii. Transects and/or distance-based methods; iii. Capture-recapture techniques; iv. Own methods developed in the study.

Considering the lack of tools for analyzing the quality of studies of the abundance of animal populations in the literature, as well as the variety of techniques used in publications, we carry out a theoretical analysis of the limitations and susceptibilities to bias in the studies reviewed. We focused on general aspects regarding the study design and execution, and the choice of an analytical methodology without building a final quality score [31]. We analyzed whether the methods utilized were appropriate for the generation of valid estimates of abundance or density of the population of dogs studied. We also analyzed the adequacy of the description of the information, the methods of estimation, the data collection procedures during field work and the form of identification and registration of dogs as well as other specific biases or limitations identified in each study. All quality analysis were performed based on concepts discussed by Williams et al. [22], Amstrup et al. [24] and Brochures [32].

As the last step in our analysis, we focused on estimates of abundance and density reported in the studies and possible patterns of results concerning the variables collected at the time of extraction.

## Results

### Geographical distribution of the studies included in the analysis

**Fig 1:**
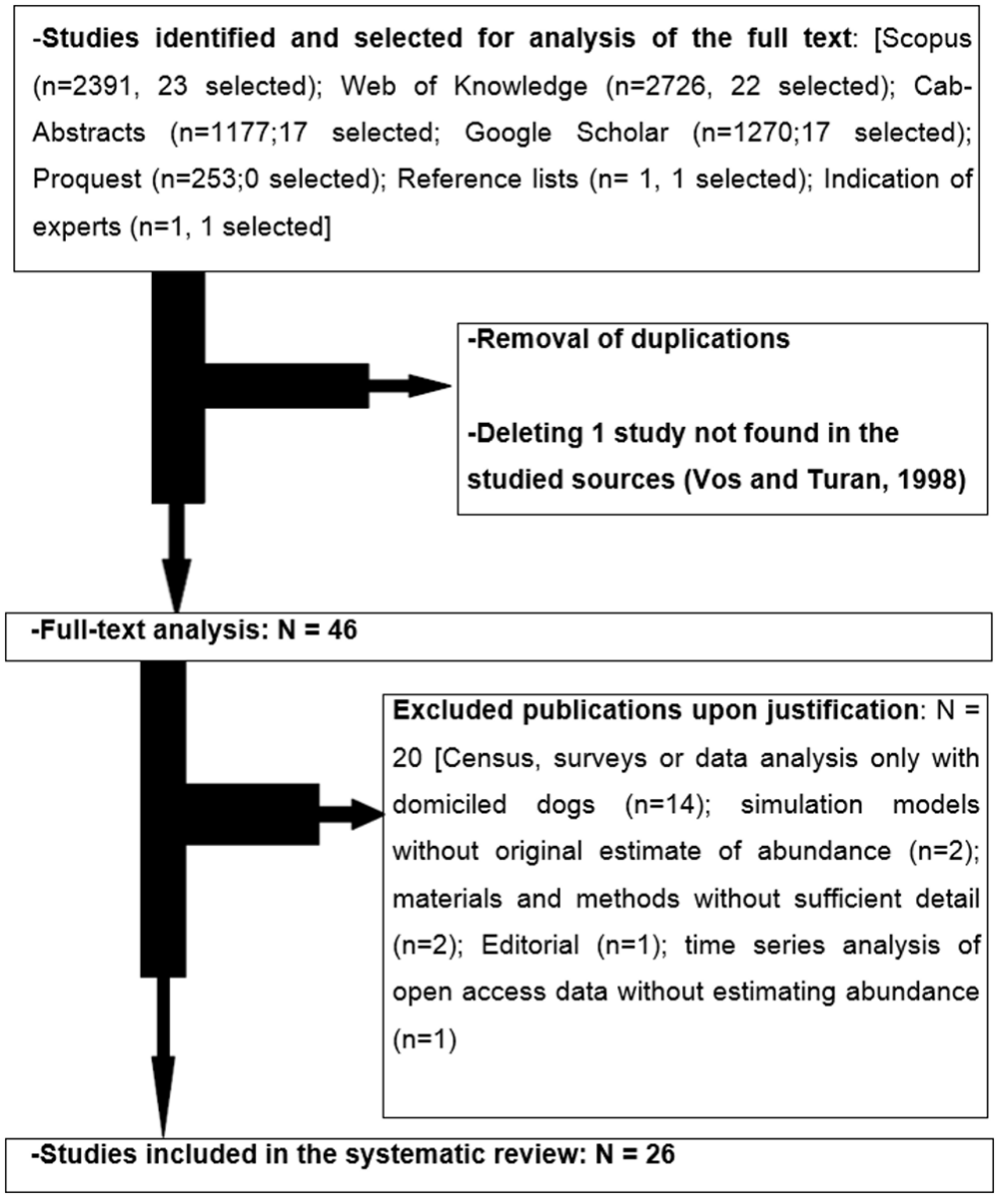
Flow chart of the selection process of the studies reviewed.

We analyzed the full text of 44 publications as the direct outcome of our searching strategy applied to the specified databases, one publication indicated by a specialist, and one recovered by the analysis of references therein. Twenty of these publications were excluded for various reasons. Thus, 26 studies were included in the final review [10, 20, 33–56] (S1 Table). Fig 1 describes the flow chart with the outcome of our searching strategy and the justifications for the exclusions performed.

Brazil was the country with the largest number of analyzed studies (n = 6), followed by India (n = 5) and Nigeria (n = 2). United States of America and other thirteen countries had only one study each. The continent with the highest number of publications was the Asian (n = 11), followed by the Americas (n = 8), Africa (n = 4) and Europe (n = 3). There were no studies performed in Oceania.

### Analysis of methods for the estimation of abundance and identification of the limitations in the implementation of studies

Seven publications reported abundance estimates based on counts; one used a method based on distances, 12 performed capture-recapture techniques and 6 used their own methods. No studies relied on statistical models to report the association of covariates of interest to abundance estimates. Table 1 describes the sources of susceptibility to bias arising from the estimation methods used in the study groups. Our analysis is stratified by those categories. S1 Table presents the details of the procedures, results and limitations of each of the studies analyzed.

**Table 1:** Main susceptibility to bias due to the methods used to estimate the abundance of the populations of free-ranging dogs.

Technique	Studies	Main potential sources of bias affecting the reported estimates*
Census surveys without corrections for different probabilities of animal detection	Aiyedun and Olugasa [33]; Berman and Dunbar [36]; Hossain et al. [44]; Ibarra et al. [45]; Ivanter and Sedova [46]; Pal [51]; Torres et al. [53]	The method does not account for possible differences in animal detection; Populations should be closed, and some studies had long execution times; Method indicated only for restricted and small populations.
Census surveys with corrections for different probabilities of animal detection	Kalati [47]	Long duration, violates the premise of closure; Method indicated only for restricted and small populations; A constant value for the probability of capture was used to correct the count. Such value was not estimated from a random sample of blocks.
Line transects	Childs et al. [37]	Measurement of the distances between the lines and the dogs may not have been adequate; Transects were not arranged randomly in the area surveyed
Lincoln-Petersen estimator	Artois et al. [34]; Shimozako and Junio [10]; Dias et al. [20]	Method does not account for potential differences in animal detection
Schumacher method	Totton et al. [54]	The method is not appropriate to estimate stray dogs; Premises required for the proper use of this approach has proven to be difficult to verify.
Method of Beck	Belsare et al. [35]; Daniels and Bekoff [38]; Faleke [39]; Font [40]; Guillloux [42]; Kato et al. [48]; Shimozako and Junio [10]	Aside from Belsare et al. (2013), studies did not account for potential heterogeneity in capture probabilities; Populations should be closed, and some studies have long execution times. Method does not account for possible differences in animal detection
Mark- resight	Punjabi et al. [52]	Possible heterogeneities were not accounted for; It was not possible to know the exact number of marked animals present in the area—premise necessary for the implementation of the adopted model (logit-normal); Monochrome dogs were considered not marked in the initial stage.
Time series analysis	Vial et al. [56]	The data collection along different years was complex and not uniform; Transects selected non-randomly; estimation by mean of counts (census) that did not account for possible differences of animal detection; possible violation of premise of closed population.
Binomial model and Bayesian analysis	Matter et al. [50]	Method dependent on the choice of the prior information.
Bayesian analysis to identify the proportion of stray dogs	Gsell et al. [41]	Method applicable only if all domiciled dogs dwell visible areas (gardens, terraces, etc.) for those who make external observations.
Extrapolation	Tung et al. [55]	Selection of points to identify dogs should be random and representative of the national geographic space, which cannot be verified; estimation by mean of counts that did not account for possible differences in animal detection
Information on dogs previously sterilized to estimate the population of strays	Hiby et al. [43]	Use of a unique survival probability value to represent the entire population; possible violation of the premise of a closed population.
Pasteur technique	Matos et al. [49]	Overlapping areas where animal counting takes place, thus dogs could have been counted more than once; study area open to immigration; Estimation based on animal counting that did not account for possible differences in animal detection; valid estimates require that all free-ranging dogs be on the streets and be identified in the short period of observations; potential information bias (see S1 Table)

* Other limitations that may render the estimates invalid such as inaccuracies in the identification of dogs and in the procedures leading to their capture or visualization have been identified in different studies and are described in S1 Table.

#### Studies using methods based on counts

Seven [33, 36, 44–46, 51, 53] of the eight studies analyzed in this group used census surveys to estimate the size of the free-ranging dog populations. These studies reported the simple counting of the dogs viewed or photographed during a given period of time, and this value was computed as the population abundance. This approach does not take into account the possibility of heterogeneous probabilities of animal detection and can only generate indicators of canine abundance instead of estimates of population parameters of abundance [22].

Animal population censuses assume closed populations along the counting period [22], which leads to the condition that the data be collected for short time periods. This latter requirement was not fulfilled in the reviewed studies where the capture period was described [33, 36, 44, 45].

Kalati [47] reported a census of the animal population in 137 blocks of the city of Kathmandu, Nepal. The count obtained at this stage was corrected by the probability of capture estimated for 16 blocks in which capture and recapture procedures had been performed over five consecutive days. While this approach might seem plausible to correct for the likelihood of animal detection in a census, it can be criticized for representing a specific subset of the 137 blocks originally sampled. Instead, a random sample of those 137 blocks would provide an unbiased estimate of the correction factor.

The studies in this group reported animal abundance estimates with statistical properties that can be regarded as questionable. Similar concerns apply to density estimates since they are simple ratios of the (inaccurately) estimated number of animals and the geographical area stipulated by authors.

The studies reviewed in this group provide incomplete descriptions of the animal capture procedures used regarding the actual path followed, the location and time of the day when the captures/observations took place, and the method used. Only studies of Torres and Prado [53] and Hossain et al. [44] described, albeit not in detail, the use of photographs for the identification of spotted dogs. Pal [51] did not describe the procedure for identification of dogs, albeit the study having been carried out between March 1994 and February 1998, with four annual capture efforts. The remaining studies recorded the physical characteristics of animals, which may be a source of inaccuracies related to the counts reported, especially in those studies [33, 36, 45] in which the number of animals was significantly high.

#### Study using methods based on distances

Childs et al. [37] estimated the density of the free-ranging dog population using a distance based method where the individuals were observed around transects located in the region of interest. Details of Childs et al. [37] study are given in S1 Table. While this technique yields a more suitable density estimate than techniques based on counts [32], it still partly relies on ad hoc methods of estimation. Childs et al. [37] adequately described the methods they used, including the animal observation technique and choice of the analytical model. However, the authors did not rely either on photos nor more sensitive ways of identifying dogs. They provided insufficient details about the way they measured the distances between the animals and the transects. Also, the transects were not randomly allocated, which violates one of the assumptions of the technique used. For logistical reasons, the transects were placed mainly on roads and highways and dogs from other areas were not represented in the sample. Although these limitations may be relevant, their prevention is difficult in these types of studies. As the authors pointed out, unrestricted dogs that do not live in any way associated with humans were extremely uncommon in the study area, which could have reduced the negative influence that the infringement of such a premise could cause.

#### Capture-recapture studies

The twelve studies using captures and recaptures assumed a closed population as a premise required by the analytical methods employed (Table 1).

Three studies [10, 20, 34] used the Lincoln Petersen estimator to infer the canine population size. This approach was used by Laplace (1786) to estimate the size of the human population in France and by Lincoln (1930) to estimate the number of waterfalls in North America [22]. It is the starting point to understand all estimators based on capture-recapture [22] and takes only two waves of capture procedures. If executed at short intervals, without incurring in losses of animal markers, and assuming homogeneous probabilities of capture [24] can produce unbiased estimates of the size of animal populations. However, the suitability of such premises cannot be directly verified, which constitutes the major limitation of the approach [57] leading to the need to increase the capture efforts [22]. The description of the procedure developed by Artois et al. [34] lacks in detail since even the interval between captures was not reported. This prevented a better assessment of their study. In the study by Shimozako and Junio [10] and Dias et al. [20], captures were performed in the morning and recaptures in the afternoon on a weekly basis in the first study and on a three months period in the second. The implementation of capture efforts and recapture on the same day limits the possibility of violation of the closure assumption of the population.

Another study that used a simple estimator based on only two efforts was Totton et al. [54], in this case, the Schumacher method, developed in 1943. We did not find references to this methodology in the tools used to analyze the quality of the studies reviewed. However, by checking the original article first describing this technique [58], we concluded that it is not appropriate to estimate populations such as dogs, since it was originally conceived for aquatic animals. Also, the premises required for the proper use of this approach has proven to be difficult to verify.

Seven studies [10, 35, 38, 39, 40, 42, 48] used the Beck method to estimate the size of the dog population. This technique, first described in 1973 [59], has the same premises as the Lincoln-Petersen estimator and extends this latter approach to k number of closed population captures using photographs for animal identification. Further advances in modeling capture and recapture data from closed populations, allowing for different assumptions about the sources of variation in capture probabilities [22, 60], have not been described in the original method by Beck [59], nor in the WHO recommendations [25]. One of the studies reviewed [35] used, in addition to Beck´s method, techniques for analyzing heterogeneity in the capture probabilities. By applying Beck´s method, the authors estimated the size of the animal population as being less than the minimum number present in the population, a quantity known to them. The long duration of the study, which jeopardizes the premise of a closed population required by Beck´s method, could explain the observed discrepancy between population size estimates. The authors then fitted a model that considered the individual capture probability, resolving this apparent discrepancy. The other publications in this review used the Beck method as recommended by the WHO and thus did not consider the possibility of bias originating from different animal capture probabilities. Such studies were also subject to violation of the closed population assumption. In fact, in 25 out of the 26 studies reviewed, the violation of this premise was not verified with the use of currently existing methods [61].

Finally, the study by Punjabi et al. [52] used Mark-resight procedures [62] to estimate the free-ranging dog population. Mark-resight models result from a slight modification of the traditional procedure of capture and recapture. This modified approach benefits from the input of additional information available obtained by the visualization of animals not previously marked [63]. Different models, each specifically tailored to the way data is collected, can be used in the analysis [62]. Considering that the photographic method enables the individual identification of each dog, it is possible to model the capture probabilities, also known as visualization probabilities. Punjabi et al. [52] assessed the presence of heterogeneity among capture probabilities. However, due to convergence problems not specified, they fitted a simpler model that did not allow for parameters describing this heterogeneity. The actual model (logit-normal) fitted by the authors did not seem appropriate once the interval between the first capture and the last observation period, 6 days, casts doubts on the actual number of marked animals present in the area, a necessary premise in their approach. The tools necessary to monitor the animals, such as radio or GPS collars that emit mortality signals, were not available in the study [64]. In any case, this period was relatively short and such bias, if present, may have had little influence on the parameter estimates. However, potential sources of biases can arise when the authors considered as not marked (in the initial stage) the monochrome dogs or dogs without the so-called natural marker. These animals were in the area and detected by the investigators; therefore, it seems more appropriate to assign these animals as marked based on other alternative physical characteristics besides the animals´ coat.

As well as in studies based on counting, the studies that performed captures and recaptures estimated density by simply dividing the estimated number of dogs by total area, which might not represent the true value of this parameter [32]. Additional limitations, often found in the studies, are the reporting of parameter estimates without the corresponding measures of uncertainty and the inadequate description of the routes, times and intervals between captures, as well as animal identification methods (S1 Table).

#### Studies that implemented their own methods

Vial et al. [56] analyzed the trend in population abundance of wandering dogs in Bale Mountains National Park, in Ethiopia, based on data collected from 1986. The data were collected at distinct time intervals throughout the different areas in the park with interruptions due to logistics problems. Animal identification and counting took place from vehicles following non-random transects. Such efforts lacked in detail and were not standardized. Taken as a whole, these issues may have rendered the study susceptible to biases and interfered with the validity of the time series estimates. Regarding the estimation procedure, it was a simple count of the observed dogs and the rate of encounters (individuals/km2). As the study areas were not closed to animal migration and the encounter probability may have changed over the years, the validity of estimates of the population density is questionable.

Matter et al. [50] implemented their own procedure to estimate canine abundance. They studied dogs marked at the time of vaccination followed by home visits and monitoring of the animal tag in the target area. Their approach relies on a binomial model of probabilities to estimate the number of unmarked dogs, the specification of a priori distributions to estimate the probability of recapture, and the Markov Chain Monte Carlo (MCMC) approach to yield the "posterior" distribution of all parameters of interest. Akin to all Bayesian approaches, their method is dependent on the choice of input prior distributions needed to run the Bayesian analysis. The authors do not offer much detail about the fieldwork leading to animal captures as well the reasons why the number of areas covered by vaccination does not overlap with the areas targeted by the household survey. Both issues represent important sources of concern.

Gsell et al. [41] tried to estimate the proportion of stray dogs also from a Bayesian perspective. Based on an innovative approach, they update estimates previously available to achieve more precise estimates of the actual population of stray dogs. In S1 Table, we offer additional details on the implementation of their work. It is noteworthy that the method developed is not robust against misclassification of the status of domestic dogs or under-reporting of wandering dogs.

In the study by Tung et al. [55], the identified dogs were counted in 56, 74 and 74 selected observation points in Taiwan respectively in 1999, 2004 and 2009. The authors estimated the national population of dogs using the following formula: [(TOTAL POPULATION OF TAIWAN–(POPULATION OF RESIDENTS IN THE AREA—TOTAL OF CAPTURED DOGS)].

We identified the following limitations in the study: due to the lack of explicit information on the selection strategy of the observation points, one cannot infer random alocation of the observation points and, therefore, a representative sample of the national geographic boundaries; the census approach presents the inadequacies described earlier in this work; there was no information on routes and the photographic procedure performed.

Hiby et al. [43] used information on the number of dogs previously sterilized by non-governmental as well as governmental organizations to estimate the population of free-ranging dogs in three cities in India. We offer additional details on how these estimates were obtained in S1 table. The approach is advantageous over alternative techniques such as simple counts. However, we emphasize that it is dependent on the availability of reliable information on the number of sterilized dogs, on estimates of the survival of these animals, and on the identification of dogs. It is also assumed that the population is closed. Shorter studies might better fulfill this latter assumption, which was not true in the present study. Also, the animal identification procedures were not adequate as well as the survival of the population of dogs at different age groups that was considered constant.

Finally, Matos et al. [49] developed what they called "Pasteur technique" to estimate the dog population in two urban areas of a Brazilian municipality. Additional details about the technique as well as important limitations that invalidate its application to estimate the size of the population of free ranging dogs are described on S1 Table.

### Parameter estimates and results of reviewed studies

Canine density indicators varied considerably among the different studies. These estimates were high in some areas of the cities of Valencia [40], Katawa [51] and Kathmandu [48], respectively 1304 dogs/km2, 1859 dogs/km2 and 2930 dogs/km2, reaching the ratio of 5.2 dogs per inhabitant in this latter city. On the other hand, in areas of the cities of São Paulo [42], Raipura [44], Mumbai [52], São Luiz do Paraitinga [53] and Jodhpur [54] density estimates were lower than 10 dogs per km^2^. In the other six studies, as well as in certain areas of those studies already mentioned, the density estimates remained between the two extremes above and the ratio of dogs to inhabitants was always less than 1 (S1 Table). Only three studies describe the specific areas where dogs concentrate. Dias et al. [20] conducted their study on a college campus and showed that regions harboring higher amounts of organic material carried a larger number of animals. Font [40], in turn, described higher concentration of dogs in areas with low socioeconomic status in Valencia, Spain, and Ivanter and Sedova [46] showed that areas with higher densities of human population also display higher densities of stray dogs in Petrozavodsk, Russia.

Regarding the distribution of the populations by gender, there was a predominance of males in all studies where this information was available. Few studies provided information on age groups and only one described the breeds of dogs studied, all mongrel (S1 Table).

## Discussion

This review analyzed the methods and results of twenty-six studies that estimated the abundance, density or the ratio of human to dogs of the free-ranging dog populations. It is difficult to indicate ideal methods to estimate the size of animal populations. The choice of an optimum strategy depends on the circumstances and the resources available in each area as well as the susceptibility to bias of each technique. The papers reviewed in this work used methods that have well-known limitations and did not incorporate alternative strategies that could lead to improved estimates of animal abundance.

Dogs are territorial animals, tend to concentrate in areas with increased availability of food and have varying patterns of behavior towards people, so their individual probabilities of being captured differ. Thus, the use of counts by mean of census would only apply if one could look at the same moment the dogs in all areas of the territory [19]. In opposition to this latter recommendation, eight studies used census to estimate abundance. On the other hand, methods based on counting individuals belonging to sampling units representing the study area, and where all dogs can be counted or the sampling fraction can be estimated, are widely described in the literature [10, 57, 65, 66] but have been used only in one study reviewed [37]. Assuming that censuses were used more often because they are faster and less costly, they do not outperform sampling methods. The latter approach is capable of generating better information, and its implementation is as complex as the census, just requiring prior planning to choose the most appropriate model [65]. Also, sampling methods are cheaper when large areas need to be covered. Therefore, such methods ought to be considered in future research and, even, in regular surveillance activities requiring estimates of the abundance and density of dogs. On the other hand, counts by mean of censuses should be carried out only in rare instances when no other alternative becomes available, for a short period and, preferably, implemented along with calibration procedures and statistical tests for the presence of heterogeneities of detection probabilities [22, 66].

Regarding the twelve studies using capture-recapture techniques, only one [35] addressed the presence of heterogeneities in the analysis. None of the studies reviewed considered the populations sampled as open, although this approach were feasible and could have provided relevant information regarding the estimates of parameters such as survival and recruitment in populations of free-roaming dogs [57, 67]. It is advisable that future capture-recapture studies take advantage of the various methods available for the analysis of open populations [22, 68]. The same advice applies to the methods of analyses of closed populations that take into account heterogeneous probabilities of animal capture [60]. In this context, we call the readers´ attention to Pollock´s Robust Design, whose methodology is described in different publications [22, 69, 70], and which incorporates the advantages of both approaches developed for open and closed populations. Although being more difficult to implement due to its complexity, this technique deserves the attention of future academic research in this field.

The vast majority of the studies followed WHO recommendations, first published in 1990 [25] and the most recent document by the World Animal Protection [26]. These publications suggested the use of census, of Lincoln Petersen estimator and of Beck´s method. Both documents highlight the assumptions and limitations of these techniques but do not refer to the alternative approaches recommended otherwise. These approaches would be useful, when the assumptions are not tenable, and when the limitations are present, and could lead to adequate estimates of the size of populations of free-roaming dogs. Parameters related to the canine population dynamics, such as abundance, can have a major impact on animal welfare, animal disease control and the monitoring of the effectiveness of population control measures [18, 19]. The importance of having access to proper estimates of these parameters should be overlooked. It is, therefore, essential that WHO updates its recommendations by describing sampling techniques based on distances or count, and methods that allow for the analysis of capture probabilities of dogs in models applicable to closed populations. Such methods do not add complexity to the fieldwork data collection process and generate more valid and reliable information. Previous training on the development of the study protocol and analysis is all that is required for their implementation. Equally important is the need to raise the awareness about methods based on open populations and their role in the estimation of survival and abundance.

More expensive or complex methods, in turn, might be difficult to implement in a more general context but are certainly useful in a research context. A short list of such methods include: spatially explicit capture-recapture [32], known-fate models with individuals carrying radio markers [71, 72], Pollock´s Robust Design and Mark-resight that make use of tools to know exactly the number of marked dogs, as well as methods developed and/or used more recently to sample animal populations other than dogs [73–76]. Methods introduced in the studies by Matter et al. [50] and Hiby et al. [43] deserve further evaluation since they can prove to be useful tools for estimating the canine abundance.

In addition to the limitations introduced by the choice of the analytical methods, a second source of concerns that might affect the studies reviewed in this work is their susceptibility to biases due to the study implementation. The approaches used may experience failure to identify the dogs due to the use of inappropriate markers or lack of photographs [77]. Different markers and the photographic method can be used with canine populations [25]. Photos in adequate numbers and in different positions for the same dog may have high sensitivity and specificity [10; 78] without the need for physical contact between the researcher and the animal. Future studies should expand the description of the photographic method to better assess their suitability and reproducibility. On the other hand, biases and limitations arising from the study implementation or the description of the capturing procedures or animal visualization can benefit from tools analogous to those developed to improve the description of the results of epidemiological studies [79]. These latter guidelines have yet to be adapted to the context of studies aimed at estimating animal populations. Once available they might help in conceiving proper studies with a transparent and standardized description of the results.

Density estimates reported in the studies reviewed in this work displayed a large variation. This statement is true even in studies performed in the same country, as it is the case of India [35, 51]. As discussed by Shimozako and Junio [10], this variability prevents that a single value of the ratio of human to dogs be extrapolated to estimate the populations of different areas. Observed differences in population abundance can be considered normal, and even expected, given that the density of dogs is strongly influenced by social, economic, demographic, environmental and cultural factors [9]. We noted, however, that the modeling procedures in all the studies reviewed did not account for additional factors possibly related to the parameters describing the dynamics of the canine population [80]. Only a few of those studies provided additional descriptive information. Such analyzes should also be implemented in future research since these factors might play a key role in understanding the canine population dynamics [22].

Male animals outnumber female animals in the population of free-roaming dogs. This statement derives from the descriptive data presented in the studies. This pattern is also observed among restricted dogs [81], and may be explained by the use of male animals as guard dogs [44] and by a higher mortality among female animals due to pregnancy and childbirth [82].

The present review is also limited in various aspects. Given the geographic diversity of the studies analyzed, our search strategy did not cover all sources of information, and differential selection of studies should be reminded. We searched different bases as well as Google Scholar to minimize this source of bias. However, studies that took place in non-academic environments may have been lost. The diagnosis of susceptibility to biases in reviews requires specific tools that are not available yet in the field of animal population dynamics. Therefore, our review should be seen as a “quality review” of a theoretical nature instead of a quantitative review aiming at more precise parameter estimates by pooling together the data published by the various studies. The successful experience in using the various tools of this type in the medical field [31] should serve as an example and guidance leading to similar initiatives in the field of Ecology.

Our review examined different studies that report estimates on the abundance or density of the population of free-ranging dogs. The validity of the estimates reported is the subject of concerns due to the limitations identified in our work. Valid estimates of abundance and density are the only way to achieve a proper understanding of the canine population dynamics, a prerequisite for the planning, execution and evaluation of control actions preserving animal welfare.

## Supporting Information

S1 PRISMA Checklist PRISMA Checklist. From: Liberati A, Altman DG, Tetzlaff J, Mulrow C, Gotzsche PC, et al. (2009) The PRISMA statement for reporting systematic reviews and meta-analyses of studies that evaluate health care interventions: explanation and elaboration. PLoS Med 6: e1000100. doi:10.1371/journal.pmed1000097.
(DOC)

S1 Table Methods, results and limitations of the studies included in the systematic review. (DOCX)

## References

[1] Krebs CJ. Ecology. New York Harper and How; 1972.

[2] Little SC, Bradshaw CJA, McMahon CR, Hindell MA. Complex interplay between intrinsic and extrinsic drivers of long-term survival trends in southern elephant seals. BMC Ecol. 2007; 7:3 17389038 10.1186/1472-6785-7-3 PMC1855316

[3] Bullock JA. Population estimation in the Torrent frog, Amolops lamtensis (Anura: Ranidae). Journal of Zoology. 1969; 159: 167–180.

[4] Manning JA, Goldberg CS. Estimating population size using capture–recapture encounter histories created from point-coordinate locations of animals. Methods Ecol Evol. 2010; 1: 389–397.

[5] Ashe E, Wray J, Picard CR, Williams R. Abundance and Survival of Pacific Humpback Whales in a Proposed Critical Habitat Area. PLoS ONE. 2013; 8: e75228 10.1371/journal.pone.0075228 24058666 PMC3772752

[6] Kirkeby C, Bødker R, Stockmarr A, Lind P, Heegaard PMH. Quantifying Dispersal of European Culicoides (Diptera: Ceratopogonidae) Vectors between Farms Using a Novel Mark-Release-Recapture Technique. PLoS One. 2013; 8: e61269 10.1371/journal.pone.0061269 23630582 PMC3632603

[7] Güthlin D, Storch I, Küchenhoff H. Toward Reliable Estimates of Abundance: Comparing Index Methods to Assess the Abundance of a Mammalian Predator. PLoS One. 2014; 9: e94537 10.1371/journal.pone.0094537 24743565 PMC3990582

[8] Massei G, Miller LA. Nonsurgical fertility control for managing free-roaming dog populations: a review of products and criteria for field applications. Theriogenology. 2013; 80: 829–838. 10.1016/j.theriogenology.2013.07.016 23998740

[9] Reece JF, Chawla SK, Hiby EF, Hiby LR. Fecundity and longevity of roaming dogs in Jaipur, India. BMC Vet Res. 2008; 31: 4–6. 10.1186/1746-6148-4-6 PMC2266723 18237372

[10] Shimozako AJ, Cout-Junior EB. Photographic capture-recapture for estimation of stray dog population. Saarbrucken Verlag; 2008.

[11] Alves AJS, Guiloux AGA, Zetun CB, Polo G, Braga GB, Panachão LI, et al Abandonment of dogs in Latin America: review of literature. Continuous Education Journal in Veterinary Medicine and Zootechny of CRMV-SP. 2013; 11: 32–39.

[12] Amaku M, Dias RA, Ferreira F. Dynamics and Control of Stray Dog Populations. Math Popul Stud. 2010; 17: 69–78.

[13] Garcia RCM, Calderón N, Ferreira F. Consolidation of international guidelines for management of canine populations in urban areas and proposed indicators for their management. Rev Panam Salud Publica. 2012; 32: 140–144. 23099875 10.1590/s1020-49892012000800008

[14] Ugbomoiko US, Ariza L, Heukelbach J. Parasites of importance for human health in Nigerian dogs: high prevalence and limited knowledge of pet owners. BMC Vet Res. 2008; 9:4:49. 10.1186/1746-6148-4-49 PMC2615757 19068110

[15] Tenzin DNK, Gyeltshen T, Firestone S, Zangmo C, Dema C, Rawang G, et al Dog Bites in Humans and Estimating Human Rabies are Mortality in Rabies Endemic Areas of Bhutan. PLoS Negl Trop Dis. 2011; 5: e1391 10.1371/journal.pntd.0001391 22132247 PMC3222627

[16] Lunney M, Jones A, Stiles E, Waltner-Toews D. Assessing human-dog conflicts in Todos Santos, Guatemala: bite incidences and public perception. Prev Vet Med. 2011; 102: 315–320. 10.1016/j.prevetmed.2011.07.017 21872951

[17] Høgåsen HR, Er C, Di Nardo A, Dalla-Villa P. Free-roaming dog populations: a cost-benefit model for different management options, applied to Abruzzo, Italy. Prev Vet Med. 2013; 112: 401–413. 10.1016/j.prevetmed.2013.07.010 23973012

[18] Dalla Villa P, Kahn S, Stuardo L, Iannetti L, Di Nardo A, Serpell JA. Free-roaming dog control among OIE countries which are members. Prev Vet Med. 2010; 97: 58–63. 10.1016/j.prevetmed.2010.07.001 20709415

[19] Fei SY, Chiang JT, Fei CY, Chou CH, Tung MC. Estimating stray dog populations with the regression method versus Beck’s method: a comparison. Environ Ecol Stat. 2012; 19: 485–498.

[20] Dias RA, Guilloux AGA, Borba MR, Guarnieri MCL, Prist R, Ferreira F, et al Size and spatial distribution of stray dog population in the University of São Paulo campus, Brazil. Prev Vet Med. 2013; 110: 263–273. 10.1016/j.prevetmed.2012.12.002 23273378

[21] Serafini CA, Rosa GA, Guimaraes AM, De Morais HA, Biondo AW. Survey of owned feline and canine populations in apartments from a neighbourhood in Curitiba, Brazil. Zoonoses Public Health. 2008, 55: 402–405. 10.1111/j.1863-2378.2008.01171.x 18811904

[22] Willians BK, Nichols JD, Conroy MJ. Analysis and Management of Animal Populations. San Diego Academic Press; 2002.

[23] Schwarz CJ, Seber GAF. Estimating animal abundance: Review III. Statistical Science. 1999; 14: 427–456.

[24] Amstrup SC, McDonald TL, Manly BFJ. Handbook of Capture-Recapture Analysis. 1st ed New Jersey Princeton University Press; 2005.

[25] World Health Organization/World Society for the Protection of Animals. Guidelines for dog population management. Geneva; 1990.

[26] World Organization for Animal Health (OIE). Terrestrial Animal Health Code, Chapter 7.7, Stray dog population control. Available: http://web.oie.int/eng/normes/mcode/en_chapitre_1.7.7.htm. 2010.

[27] Fernandez FAZ. Methods for estimating the population parameters by capture mark and recapture. Brasiliensis Ecology. 1995; 2: 01–26.

[28] Cormack R. Population Size Estimation and Capture–Recapture Methods. International Encyclopedia of the Social & Behavioral Sciences 2002; 11809–11813.

[29] Pledger S, Pollock KH, Norris JL. Open Capture-Recapture Models with Heterogeneity: II. Jolly-Seber Model. Biometrics. 2009; 59: 786–794. 10.1111/j.0006-341x.2003.00092.x 14969456

[30] Downes MJ, Dean RS, Stavisky JH, Adams VJ, Grindlay DJ, Brennan ML. Methods used to estimate the size of the owned cat and dog population: a systematic review. BMC Vet Res. 2013; 9:121 10.1186/1746-6148-9-121 23777563 PMC3689088

[31] Sanderson S, Tatt ID, Higgins JP. Tools for assessing quality and susceptibility to bias in observational studies in epidemiology: a systematic review and annotated bibliography. Int J Epidemiol. 2007; 36: 666–676. 17470488 10.1093/ije/dym018

[32] Brochers D. A non-technical overview of spatially explicit capture–recapture models. J Ornithol. 2010; 152 (Suppl 2): S435–S444.

[33] Aiyedun JO, Olugasa BO. Use of aerial photograph to enhance dog population census in Ilorin, Nigeria. Sokoto Journal of Veterinary Sciences. 2012; 10: 22–27.

[34] Artois M, Osman F, Kilani M, Wandeler A. New contribution to the knowledge of the ecology of stray dogs in Tunisia. Comp Immunol Microbiol Infect Dis. 1986; 9: 4–5.

[35] Belsare AV, Gompper ME. Assessing demographic and epidemiologic parameters of rural dog populations in India during mass vaccination campaigns. Prev Vet Med. 2013; 111: 139–146. 10.1016/j.prevetmed.2013.04.003 23664490

[36] Berman M, Dunbar I. The social behaviour of free-ranging suburban dogs. Applied Animal Ethology. 1983; 10: 5–17.

[37] Childs JE, Robinson LE, Sadek R, Madden A, Miranda ME, Miranda NL. Density estimates of rural dog populations and an assessment of marking methods during a rabies vaccination campaign in the Philippines. Prev Vet Med. 1998; 33: 207–218. 9500175 10.1016/s0167-5877(97)00039-1

[38] Daniels TJ, Bekoff M. Population and social biology of free-ranging dogs, *Canis familiaris* . J Mammal. 1989; 70: 754–762.

[39] Faleke O. Studies on dog population and its implication for rabies control. Nigerian Journal of Animal Production. 2003; 30: 242–245.

[40] Font E. Spacing and social organization: Urban stray dogs revisited. Applied Science of Animal Behavior. 1987; 17: 319–328.

[41] Gsell AS, Knobel DL, Kazwala RR, Vounatsou P, Zinsstag J. Domestic dog demographic structure and dynamics relevant to rabies control planning in urban areas in Africa: the case of Iringa, Tanzania. BMC Vet Res. 2012; 8: 1–10. 23217194 10.1186/1746-6148-8-236 PMC3534358

[42] Guilloux AGA. Estimation of stray dog's population and its association with socioeconomics and environmental factors. M.Sc. Thesis, Faculdade de Medicina Veterinaria e Zootecnia, Universidade de Sao Paulo. 2011. Available: http://www.teses.usp.br/teses/disponiveis/10/10134/tde-07082012-181835/fr.php.

[43] Hiby LR, Reece JF, Wright R, Jaisinghani R, Singh B, Hiby EF. A mark-resight survey method to estimate the roaming dog population in three cities in Rajasthan, India. BMC Vet Res. 2011; 7. 10.1186/1746-6148-7-46 PMC3163189 21834979

[44] Hossain M, Ahmed K, Marma ASP, Hossain S, Ali MA, Shamsuzzaman AM, et al A survey of the dog population in rural Bangladesh. Prev Vet Med. 2013; 111: 134–138. 10.1016/j.prevetmed.2013.03.008 23590964

[45] Ibarra L, Fabian Espinola Q, Echeverria L. A survey to the population of existing dogs in the streets of Santiago, Chile. Avances en Ciencias Veterinarias. 2006; 21: 33–39.

[46] Ivanter EV, Sedova NA. Ecological monitoring of urban groups of stray dogs: An example of the city of Petrozavodsk. Russian Journal of Ecology. 2008; 39: 105–110.

[47] Kalati K. Street dog population survey, Kathmandu: Final Report to WSP; 2010.

[48] Kato M, Yamamoto H, Inukai Y, Kira S. Survey of the Stray Dog Population and the Health Education Program on the Prevention of Dog Bites and Dog-Acquired Infections: A Comparative Study in Nepal and Okayama Prefecture, Japan. Acta Med Okayama. 2003; 57: 261–266. 14679405 10.18926/AMO/32829

[49] Matos MR, Alves MCGP, Reichmann MLAB, Dominguez MHS. Sao Paulo Pasteur Institute technique for estimating a canine population. Cad Saude Publica. 2002; 18:1423–1428. 12244375 10.1590/s0102-311x2002000500035

[50] Matter HC, Wandeler AI, Neuenschwander BE, Harischandra L, Meslin FX. Study of the dog population and the rabies control activities in the Mirigama area of Sri Lanka. Acta Trop. 2000; 75: 95–108. 10708011 10.1016/s0001-706x(99)00085-6

[51] Pal SK. Population ecology of free-ranging urban dogs in West Bengal, India. Acta Theriol (Warsz). 2001; 46: 69–78.

[52] Punjabi GA, Vidya A, Linnell JDC. Using natural marks to estimate free-ranging dog Canis familiaris abundance in a MARK-RESIGHT framework in suburban Mumbai, India. Trop Conserv Sci. 2012; 5: 510–520.

[53] Torres PC, Prado PI. Domestic dogs in a fragmented landscape in the Brazilian Atlantic Forest: Abundance, habitat use and caring by owners. Braz J Biol. 2010; 70: 987–994. 21180903 10.1590/s1519-69842010000500010

[54] Totton SC, Wandeler AI, Zinsstag J, Bauch CT, Ribble CS, Rosatte RC, et al Stray dog population demographics in Jodhpur, India following a population control/rabies vaccination program. Prev Vet Med. 2010; 97: 51–57. 10.1016/j.prevetmed.2010.07.009 20696487

[55] Tung M, Fei C, Chiang J, Chou C, Yeh L, Liao C, et al Surveys of dog populations in Taiwan from 1999 to 2009. J. Chin Soc Anim Sci. 2010; 39: 175–188.

[56] Vial F, Sillero-Zubiri C, Marino J, Haydon DT, Macdonald DW. An analysis of long-term trends in the abundance of domestic livestock and free-roaming dogs in the Bale Mountains National Park, Ethiopia. Afr J Ecol. 2011; 49: 91–102.

[57] Sutherland WJ. Ecological Census Techniques a handbook. New York Cambridge University Press; 2006.

[58] Schumacher FX, Eschmeyer RW. The estimate of fish population in lakes or ponds. J Tenn Acad Sci. 1942; 17: 228–249.

[59] Beck AM. The Ecology of Stray Dogs: A Study of Free-Ranging Urban Animals. Baltmore York Press; 1973.

[60] Pledger S. Unified maximum likelihood estimates for closed capture-recapture models using mixtures. Biometrics. 2000; 56: 434–442. 10877301 10.1111/j.0006-341x.2000.00434.x

[61] Stanley TR, Burnham KP. A closure test for time specific capture-recapture data. Environ Ecol Stat. 1999; 6: 197–209.

[62] McClintock BT, White GC. A less field-intensive robust design for estimating demographic parameters with mark-resight data. Ecology. 2009; 90: 313–320. 19323213 10.1890/08-0973.1

[63] White GC, Shenk TM. Population estimation with radio-marked animals In Radio Tracking and Animal Populations. San Diego Academic Press; 2009.

[64] McClintock BT, White GC, Burnham KP, Pryde MA. A generalized mixed effects model of abundance for mark-resight data when sampling is without replacement In Modeling Demographic Processes in Marked Populations. New York Springer; 2009.

[65] Buckland ST, Anderson DR, Burnham KP, Laake JL. Distance sampling: Estimation of Biological Populations. New York Chapman and Hall; 1993.

[66] Nichols JD, Hines JE, Sauer JR, Fallon FW, Fallon JE, Heglund PJ. A double observer approach for estimating detection probability and abundance from point counts. Auk. 2000; 117: 393–408.

[67] Cowen LL, Schwarz CJ. The Jolly-Seber model with tag-loss. Biometrics. 2006; 62: 699–705. 16984310 10.1111/j.1541-0420.2006.00523.x

[68] Schwarz CJ. The Jolly-Seber Model: More Than Just Abundance. J Agric Biol Environ Stat. 2001; 6: 195–205.

[69] Pollock KH. A capture—recapture designed to robust to unequal probability of capture. Journal of Wildlife Management. 1982; 46:757–760.

[70] Kendall WL, Nichols JD. Estimating state-transition probabilities for unobservable states using capture-recapture/resighting data. Ecology. 2002; 83: 3276–3284.

[71] Johnson HE, Mills LS, Wehausen JD, Stephenson T R. Combining ground count, telemetry, and mark–resight data to infer population dynamics in an endangered species. J Appl Ecol. 2010; 47: 1083–1093.

[72] Mills LS. Conservation of Wildlife Populations: Demography, Genetics, and Management. Hoboken Wiley-Blackwell; 2012.

[73] Huggins R. On the use of linear models in the estimation of the size of a population using capture–recapture data. Stat Probab Lett. 2007; 77: 649–653.

[74] Royle JA, Nichols JD, Karanth KU, Gopalaswamy AM. A hierarchical model for estimating density in camera-trap studies. J Appl Ecol. 2009; 46: 118–127.

[75] Kery M, Schaub M. Bayesian Population Analysis using WinBUGS: A hierarchical perspective. San Diego Academic Press; 2011.

[76] Sawaya MA, Stetz JB, Clevenger AP, Gibeau ML, Kalinowski ST. Estimating Grizzly and Black Bear Population Abundance and Trend in Banff National Park Using Noninvasive Genetic Sampling. PLoS One. 2012; 7: e34777 10.1371/journal.pone.0034777 22567089 PMC3342321

[77] McDonald TL, Amstrup SC, Manly BFJ. Tag loss can bias Jolly-Seber capture–recapture estimates. Wildlife Society Bulletin. 2003; 31: 814–822.

[78] Skrzypczak U. Wildlife photography: on safari with your DSLR: equipment, techniques, workflow. Santa Barbara Rockynook; 2010.

[79] Vandenbroucke JP, von Elm E, Altman DG, Gotzsche PC, Mulrow CD, Pocock SJ, et al Strengthening the Reporting of Observational Studies in Epidemiology (STROBE): explanation and elaboration. PLoS Med. 2007; 4:e297 17941715 10.1371/journal.pmed.0040297 PMC2020496

[80] Burnham K, Anderson D. Model Selection and Multi-Model Inference. New York Spring-Verlag; 2002.

[81] Margawani KR, Robertson ID. A survey of urban pet ownership in Bali. Veterinary Records. 1995; 137: 486–488. 10.1136/vr.137.19.486 8578662

[82] Belo VS, Struchiner CJ, Werneck GL, Barbosa DS, Oliveira RB, Neto RGT, da Silva ES. A systematic review and meta-analysis of the factors associated with *Leishmania infantum* infection in dogs in Brazil. Vet Parasitology. 2013; 195: 1–1. 10.1016/j.vetpar.2013.03.010 23561325

