# Supplementary material for: Population Estimation Methods for Free-Ranging Dogs: A Systematic Review
Source: PLoS One. 2015 Dec 16;10(12):e0144830. doi: 10.1371/journal.pone.0144830 (PMC4684217; doi:10.1371/journal.pone.0144830)
Supplement: S1 Table — (DOCX) [file pone.0144830.s002.docx]

**S1 Table. Methods, results and limitations of the studies included in the systematic review**

| **Study / Place of execution** | **Definition of free-roaming dogs** | **Form of identification of dogs** | **Methods of capture or observation** | **Method to estimate the population size** | **Abundance and/or density** | **Population characteristics** | **limitations** |
| --- | --- | --- | --- | --- | --- | --- | --- |
| Aiyedun and Olugasa 2012 [33] Ilorin, Nigeria | Counting of all dogs in the area. Classification in categories domiciled or stray from interviews with residents | Visualization and identification of physical characteristics | Census - Five researchers roamed county areas during four months - the procedure was repeated 3 times in 1 year. | Each dog found inside or outside the household was counted | First census: 3004; Second: 2800; third, 2704. | X | - Identification by inspection –Censuses by observing only one point in time on every street.  - Three Separate periods of observation and the analysis was done independently. The questionnaire method was not detailed - Period of captures not described |
| Artois et al., 1986 [34] Zaghouan, Tunisia | Inaccurate definition, all dogs assumed as stray animals. | Nylon collars | Dogs marked in a canine rabies vaccination campaign, tracked in a second time | Lincoln Petersen | 1 dog for every 6.8 inhabitants of the area | 2.75 males for each female; 0.24 young dogs for each adult | -No details of the methods of capture and marking -First marking in a vaccination campaign, thus, not random – density estimate is subject to uncertainty |
| Belsare et al., 2013 [35] 6 villages in Nannaj, Maharashtra, India | Dogs circulating in the areas traveled by vehicles | Photographic identification | Captures between 7am - 9 am and 5pm - 7 pm. In every six villages, 5 independent captures were made. It is also recorded the number of dogs vaccinated in previous campaigns | 3 methods: i. with data obtained in vaccination campaigns, the minimum number of dogs present in the area was registered; ii. Beck method; iii. Mh model with the Jacknife estimator. | Estimates by the Beck method were negatively biased, and less than the known number of animals in the area. Therefore, only the results of the model Mh were described. Median of 134 dogs in the six villages (90-188); Density of 719 dogs per km2; 1 dog to 34 (30-47) people. | X | It is said that the visualizations were daily and that 5 were made, but in another passage it is stated that some areas were covered in 3 days –Density estimated is subject to uncertainty |
| Berman and Dunbar, 1983 [36]  Residential area of Berkeley, California, United States | Dogs seen circulating in the study areas or seen in private homes without restriction | Not described | The observation area was covered 105 times by a vehicle between April and October 1977. | Counting of identified dogs | 1243 dogs | X | Long periods of counts without detailing the methodology; Procedure for identification of dogs is not described - long time and possible violation of closed population assumption |
| Childs et al., 1997 [37] 30 villages of Sorsogon Province, Philippines | Dogs seen circulating in the study areas | Visualization and identification of physical characteristics of dogs | 55 transects were mainly placed around public roads or highways. Perpendicular distances to the dog measured with a nylon tape. Transects were covered on foot or with vehicles. | Models fitted with the software DISTANCE. The hazard rate model with the distribution of distances truncated in 38,7m in 11 sample intervals was the best model. 1034 observations of dogs or groups. | 468.4 dogs per km2 (95% I.c 358.9-611.2). | X | There may have been biases in the measurement of distances; Transects not randomly arranged leading to less than optimal representation of the population |
| Daniels and Bekoff, 1999 [38] Juarez in Mexico (two regions of an urban area) and Navajo reserve | Dogs circulating in the capture area | Sightings, notes, and photos | Same route traveled by automotive vehicles for five consecutive mornings | Modified Beck method (Schnabel, 1938) | 376 (+ - 53) dogs in site 1 Juarez and 556 (+ - 70) at Site 2; 228 (+ - 29), and 431 (+ -56) in areas classified as rural; 534 dogs by km² in site 1 Juarez and 936 per km² in site 2. For the rural areas the densities are not reported | 1.6 and 2 males for each female, respectively in the two urban sites; 4 and 3 males for each female in the rural sites l; puppies and juveniles corresponded between 7 and 9% of the population in urban sites 1 and 2, densities are not reported for the remaining sites | - Beck Method that does not account for possible differences in animal detection – Density estimated is only an indicator; No details about the capturing procedures |
| Dias et al., 2012 [20] São Paulo University campus, Brazil | Dogs circulating on campus | Sightings, notes, and photos | Five capture efforts with three-month intervals - except for the two months of vacation. Circulation by car or on foot, held in the morning and afternoon of each day | Five estimates were made (one for each capture effort, considering dogs seen in the morning and reviewed in the afternoon). Lincoln Petersen estimator | Population size in the five captures and recaptures varied between 36 and 52 | Around 1.5 male for each female considering all captures; areas with organic matter concentration such as restaurants in the University had a bigger number of dogs | Individual abundance estimates were calculated using a simple estimator. The complete history was not analyzed to identify possible heterogeneities. |
| Faleke, 2003 [39] 4 areas in Agbowo, suburban area of Ibadan, Nigeria | Dogs circulation in the selected capture areas | Sighting | Captures in streets of the 4 areas. Procedure repeated daily during 20 days in each area between 6am and 7am | Modified Beck method (Okoh, 1988) | 69, 108, 86 and 75 dogs in each one of the areas, 1 dog for 11 people | X | No details of the capture procedure – Identification by sighting – estimated density is only an indicator – long time and possible violation of the assumption of closed population – Beck method without considering possible heterogeneities |
| Font, 1987 [40] Seven areas of Valencia, Spain | Dogs circulation in the selected capture areas | Photos | Each area (called transect) was covered by 9 consecutive days using a motorcycle | Beck method | Between 127 and 1304 dogs/km² in the seven areas | Two males for each female; Bigger densities in areas with worse socioeconomic conditions | - Beck Method without considering possible heterogeneities - Estimated density is only an indicator; No details about the capture procedures |
| Gesell et al., 2012 [41] Four regions of the Iringa county, Tanzania | The study tried to identify the proportion of actual stray dogs in a population. This population consisted of domiciled and semi domiciled dogs. Those without any supervision were considered stray | Visualization of collars applied in the first stage of the study - a vaccination campaign with the subsequent active search for animals in residences of the study area. | Study stages: 1-Census covering all houses to count the dogs; 2- Collection of a sample with application of a detailed questionnaire, checking, among other information, the confinement of the animals; 3- Marking of vaccinated dogs and active search; 4- Recaptures: circulation of cars for two days in two periods in three transects that covered 5% of the area. Notes were taken on the degree of restriction | In procedure 4, some of the visualized animals had a collar (i.e. they were domiciled, vaccinated and marked) and others did not (they could be domiciled, but non-vaccinated, or they might have lost the collar or were in fact stray dogs). Thus, in stage 2 there was information about the confinement of the animals. Through Bayesian remodeling, the proportion of dogs that were, in fact, stray in the population was estimated | In the census, 2498 dogs were identified. Less than 1% of the population was considered wandering | 58% of dogs were male | Premise that domiciled dogs were visible in step 4 is not possible in most circumstances, which may impair the application of the method |
| Guillloux, 2011 [42] 6 areas in São Paulo city, Brazil | Dogs circulating in the selected capture areas | View, photographs, and notes | Two days of catches with two captures each day - in the morning and in the afternoon. Route by car or on foot. | Among the six areas, only two had dogs: Garden Cybele = 96 dogs & Assumption = 12 dogs; Density of 3.37 (Assumption) and 40.68 per km of streets | Beck method | X | Beck method without considering possible heterogeneities |
| Hiby et al., 2011 [43] 3 cities (Jaipur, Jodhpur, and Jaisalmer) in the state of Rajasthan, India | Dogs circulating in the study area | The information about the dogs previously sterilized by organizations and public authorities was used. All dogs received a cut on the ear during sterilization and were easily identifiable | Procedures were guided by observing the number of dogs that were marked and the number of dogs that were not. Locomotion by vehicles or on foot in a pre-defined route. In each of the three cities, there were slight differences in the procedure. | A Mt model was used for estimating the number of dogs using the data of the sterilized ones. The following calculation was performed: total number of dogs = R; number of surviving dogs sterilized at that point of time = r; p was the percentage of dogs in a random sample and equal to 100r / R. The survival of marked dogs was estimated using data from previous studies (0.70 per year). | 36,580 dogs in Jaipur (6,562 to 46,597). 24,853 in Jodhpur (18,364 to 31,341). 2,962 in Jaisalmer (1,721 to 4,202) | X | Dog survival information is scarce in the literature, and the model needs accurate survival estimates to be valid. Only one value of survival may not accurately reflect the population structure - Possible failure to identify dogs / Different dogs sighting probabilities |
| Hossain et al., 2013 [44] Six regions in the Narsingti county, Raitura, Bangladesh | Dogs seen in the study area | Photographic method | From October to December 2010 volunteers and researchers traveled around the streets of the studied areas | Counting the number of dogs seen | 461 dogs; 14 (3.7 - 24.) dogs/km²; 1 dog for 120 people | 570 males, 290 (25%) females and 283 with undetermined genre | Few details about the methodology; Density estimated is only an approximation; Census was executed by many volunteers that may have affected the validity |
| Ibarra et al., 2006 [45] 25 randomly selected blocks in 34 communities belonging to the province of Santiago, Chile | Dogs seen circulating the study area | Sighting | Observation held for an hour on every street | Counting of the number of sighted dogs | 5666 dogs | 70,1% male 21,6% female and 8,3% with undetermined gender | - Large number of dogs and Identification by viewing - Census with only one observation on each street. |
| Ivanter and Sedova, 2003 [46] 11 areas in the city of Petrozavodsk, Russia | Dogs sighted circulating the study area | Photographic method | Annual data collections from 2002 to 2007 - three times every season | Counting of the number of sighted dogs | In average, 1271 dogs per year; 36,8/km² | 56% male and 46% female. Areas with a bigger population density had more dogs | Few details about the methodology. It was not informed if the teams were the same, if they had been trained and if there were standardization; Estimated density is only an approximation; Census without considering possible heterogeneities. |
| Kalati, 2010 [47] 137 blocks selected randomly in Kathmandu, Nepal (in 16 were executed capture-recapture procedures) | Dogs sighted circulating the study area | Photographic method | Initially a census for counting the dogs in all blocks. To estimate capture probabilities, capture-recapture: 5 efforts (on consecutive days) | Estimated capture probability was used to correct the counts of 137 blocks | 22,555 ± 2561 dogs; 5.245 ± 0.595 dogs/hectare | 1.31 male for each female | A single capture probability value was used (average in blocks) to correct the counts of areas and dogs with different characteristics. - The modeling process for selecting the Mh model in capture-recapture estimation was not described / Density is only an approximation; Census without considering possible heterogeneities |
| Kato et al., 2003 [48] Two delimited areas in two cities: Kathmandu in Nepal & Shimotsui, in Japan | Dogs sighted circulating the study area | Photographic method | Four capture efforts. Walking routes between 5:30 am 6:30 a.m. | Beck method | 17.4 dogs in Kathmandu and 1.4 dogs in Shimotsui; 2930 dogs per Km² in Kathmandu and 225 per Km² in Shimotsui; 1.47 for each person in Kathmandu and 1: 5.2 in Shimotsui | X | Beck method without considering possible heterogeneities – Estimated density is only an approximation |
| Matos et al., 2002 [49] Two urban sectors of Serra Azul County, Brazil | The study used a method developed by the authors to estimate the dog population of the area, classifying dogs into 4 types: restricted; with owners but not restricted; neighborhood; wandering (without owners). | In home visits, restricted dogs and that with owners but not restricted received collars of different colors. Neighborhood dogs received another kind – collar put in place by the resident who claimed the dog. Stray dogs did not receive collars. Collars of a fourth color were given to residents of the surrounding area | Households in the area were visited to obtain information about the dogs and their habits (colored collars). The counting of stray dog was taken on the next day. For this, the researchers were positioned throughout the area (in non-overlapping regions), in locations that allowed the vision of collars. The counts were performed for one hour, repeating every 15 minutes | Counting of dogs in each category (survey data plus observations in the street). Among the various counts, the one with the largest number of dogs in each stratum was used. | 103 dogs roaming the streets were observed. The dog population was estimated at 498 animals | X | Dogs may have been counted more than once, since the investigators did not have overlapping areas, but the dogs could move - Dogs from other areas may not have been given collars and have entered in the counting - Wrong information from owners (noted by authors with regard to the restriction) may invalidate the method – Design to estimate abundance is actually a simple count / The single premise of the technique is unlikely (all stray dogs must be on the streets at the time of capture) |
| Matter et al., 2000 [50] Two rural areas of Gampaha, Sri Lanka | Dogs that had free access to streets. Stratification in different categories | Sighting of the collars previously put in the animals | Initially in a vaccination campaign, the dogs led by owners were marked (collars of 12 different colors). Over the next four days, the household survey with owners to check the status of marking of dogs took place. In a last step, the study area was covered 3 times for the identification of the dogs marked in each category | The number of unmarked dogs was estimated by binomial probability model. Different prior values were assigned for estimating the probabilities of recapture for the different classes of dogs. Finally, the Markov chain by Monte Carlo was applied | The binomial model estimated a count of 632 (579, 694) dogs in area 1 and 478 (423, 543) in area 2 and 1109 for the area as a whole. Ownerless dogs represented 15.5% (8.3, 23.1%) of the animals in area 1, 24.3% (14.6, 34.2%) in area 2, and 19.3% (13.5, 25.4%) for the entire region. The subsequent estimates for the total number of stray dogs was 737 (66.4, 60.9, 71.6%) | 60% male | -There were twelve areas of vaccination and eleven under investigation -No details of the capture procedures in step 3 - Dependence on the assumed premises and priors for Bayesian analysis,- Categories restricted dogs and dogs with owner had no plausibility |
| Pal, 2001 [51] Delimited area of Katawa, India | Dogs sighted circulating the study area | No description | The study was developed between March 1994 and February 1998 with 4 annual efforts of capture. Fixed routes were traveled on foot or by bike | Counting of dogs in each effort | 280 individual dogs registered in the 4 years. Variation between 150 and 200; 1859 +- 19 per Km² | 1.37 male for each female | - Long-term census without details on how it was performed, whether teams were the same and how they were trained – the procedures to identify the dogs were not described |
| Punjab et al., 2012 [52] Area in the suburb of Mumbai, India | Dogs sighted circulating the study area. In the initial stage (regarded as marking), only marked dogs with physical characteristics that distinguish them were considered | Photographic method | Initially, the area was covered with a motorcycle. In an initial step (considered as marking), only marked dogs with physical characteristics that distinguished them (called natural markers) were considered marked. Two days later and for three consecutive days viewing procedures were performed. Dogs with no natural markers were considered unmarked. | Mark Resight. Estimation by logit model - normal (after selection procedures based on AIC criteria). Models considering individual heterogeneities were adjusted, but, according to the authors, there was no convergence in the estimation | 680.64 ± 34.06 dogs (95% CI = 617.22 – 751.35) | X | Due to the analytical procedure, it was not possible to check for individual heterogeneity; The number of marked animals actually present in the area in viewing periods was not known. This information was a necessary precondition for the application of the adopted model (logit - normal); Monochrome dogs were considered not marked in the initial stage. Although this decision facilitates the logistics of the efforts, monochrome dogs were present in the area and should have been considered marked. With a photographic method, it would be possible to differentiate them by the observation of physical characteristics other than their coat |
| Shimozako and Junio, 2008 [10] A district of Ibiuna, Brazil | Dogs sighted circulating the study area | Photographic method | Three days of captures, one per week (sequentially). Each day captures were performed in the morning and in the afternoon. | Estimates were obtained considering each capture shift as a single effort, or by grouping each day as a single effort. Methods used: Peterson, Chaperman, and Beck | Quantity was around 70 dogs in the different analysis | X | None of the methods considers possible heterogeneities in the capture probabilities |
| Torres and Prado, 2010 [53] Two rural areas of São Luiz do Paraitinga, Brazil | Dogs sighted circulating the study area | Photographic method | A total of 42 km of roads (half of all areas) were covered in the morning for two days, twice. One effort each year (2004 and 2005). | Counting of photographed dogs | X | 5.7 ind/km² (Area, 1) and 6.9 ind/km² (Area 2) | Few details about the paths - Only half of the area was covered; Density estimated is only an approximation; Census without considering possible heterogeneities in the capture probabilities |
| Totton et al., 2012 [54] Six different areas of Jodhpur, India | Dogs sighted circulating the study area | Marking using colored sprays | Two periods: 2005 and 2007. In each of them, the capture methodology was the same. Captures for 5 days, between 8am and 10am. | Schumacher method (1943) | The conditions for the use of the estimator were violated in the area 1, so the estimates were given for areas 2-6 separately. In 2005, the area 1 had 463 dogs, the other ones ranged from 112 to 189. In 2007, the area 1 had 126, and the others ranged 68-113. | 1.3 and 1.4 for each female in 2005 and 2007 respectively | Method developed for populations with different characteristics - There may have had lost markers - Confusing methodology for verifying model assumptions |
| Tung et al., 2010 [55] Respectively, 56, 74 and 74 points of observation selected randomly in the years 1999, 2004 and 2009 in Taiwan. | Dogs sighted circulating the study area | Photographic method | In the three periods (years) of observation, the area was covered by bikes. The number of dogs photographed was registered | Given i. the number of photographed dogs; ii. The number of residents and; iii. the total population of Taiwan, the national dog population was calculated by the division: [(total population of Taiwan) / (population living in the areas / total captured dogs)] | In the first effort, 3756 dogs were identified, in the second 2137 and in the third 1429. Estimates of dogs, respectively in the three periods using the proposed quotient: 613.959 -120.476 - 86.244 | X | - Points were not selected according to a standard practice in the three periods and were not representative of the national geographic space -Few details on the method. The standardization procedures were not described as well as whether the teams remained the same, and how they were trained – Census without considering possible heterogeneities in the capture probabilities |
| Vial et al., 2010 [56] Bale Mountains National Park, region of Oromia, Ethiopia | Dogs sighted circulating the study area | Photographic method | Data collected between 1986 and 2007 in the park by the Ethiopian Wolves Conservation Program. Captures in a partitioned transect in three areas. Counts were conducted in a vehicle for 2-4 observers. Each year, different amounts of effort were made; no activity between 1993 and 1995 | Encounter rates (individuals/km) per year and habitat pondered by the station | Values varying in the periods and stations, with a minimum of zero and a maximum of four dogs/km | X | Few details on the methodology. The standardization procedures were not described as well as whether the teams remained the same, and how they were trained - Data collection activities subject to interruptions and different number of executions along the year- Census - without considering possible heterogeneities in the capture probabilities density estimated is just an approximation |
